# A cross-sectional study of food group intake and C-reactive protein among children

**DOI:** 10.1186/1743-7075-6-40

**Published:** 2009-10-12

**Authors:** M Mustafa Qureshi, Martha R Singer, Lynn L Moore

**Affiliations:** 1 Section of Preventive Medicine and Epidemiology, Boston University School of Medicine, Harrison Court, 761 Harrison Ave, Boston, MA 02118, USA

## Abstract

**Background:**

C-reactive protein (CRP), a marker of sub-clinical inflammation, is a predictor of future cardiovascular diseases. Dietary habits affect serum CRP level however the relationship between consumption of individual food groups and CRP levels has not been established.

**Methods:**

This study was designed to explore the relation between food intake and CRP levels in children using data from the cross-sectional 1999-2002 National Health and Nutrition Examination Surveys. CRP level was classified as low, average or high (<1.0, 1.0-3.0, and >3.0 mg/L, respectively). Adjusted mean daily intakes of dairy, grains, fruit, vegetables, and meat/other proteins in each CRP category were estimated using multivariate analysis of covariance modeling. The effect modification by age (5-11 years vs. 12-16 years), gender and race/ethnicity was explored. We examined whether total or central body fat (using BMI Z-scores and waist circumference) explained any of the observed associations.

**Results:**

A total of 4,010 children and adolescents had complete information on diet, CRP and all covariates of interest and were included in the analyses. Individuals with high CRP levels had significantly lower intake of grains (p < 0.001) and vegetables (p = 0.0002). Selected individual food subgroups (e.g., fluid milk and "citrus, melon and berry" consumption) were more strongly associated with lower CRP than were their respective major food groups. Consumption of meat/other proteins did not influence CRP levels. The addition of body composition variables to the models attenuated the results for all food groups to varying degrees.

**Conclusion:**

Children and adolescents with higher CRP levels had significantly lower intakes of grains and vegetables. The associations between selected childhood dietary patterns and CRP levels seem largely mediated through effects on body composition.

## Background

C-reactive protein (CRP), an acute phase protein, is a non specific biomarker of inflammation produced by the liver in response to various inflammatory conditions [1]. The synthesis of CRP is regulated by cytokines, for the most part Interleukin-6 (IL-6) [2]. The development of highly sensitive CRP assays (hs-CRP) has enabled the identification of more subtle elevations in CRP that may predict the increased risk of atherogenic cardiovascular diseases (CVD)[3,4] with better specificity than traditional risk factors such as low-density lipoprotein (LDL) cholesterol levels[4].

Sub-clinical inflammation, as measured by CRP, has been independently associated with fasting insulin levels in overweight children and adolescents [5]. Thus these inflammatory markers may be important early predictors of adult chronic disease risk and may be pro-atherogenic even at that early stage of life [6].

A number of studies have analyzed the relationship between dietary factors and CRP in adults. For example, higher intakes of grains, fruits and vegetables [7,8] in adults have been associated with lower CRP levels. Similarly, those following or adopting a Mediterranean-style diet [9,10] had reduced levels of inflammation. Among children, there are few data on the effects of diet on inflammatory markers although dietary fat (but not consumption of milk and meat) was positively associated with CRP levels in 6-14 year-old children[11]. One intervention that combined activity and a diet rich in unrefined carbohydrates, fiber (from grains, fruits and vegetables), protein-based foods and low-fat dairy products led to a lowering of CRP levels in obese children[12].

The goal of this study is to evaluate the association between the intake of foods in each of the United States Department of Agriculture (USDA) major Food Pyramid serving groups[13] and serum CRP levels in children (ages 5-16 years). Data from the cross-sectional 1999-2002 National Health and Nutrition Examination Surveys (NHANES) will be used to address this question.

## Methods

### Study population

The NHANES are cross-sectional population-based surveys conducted by National Center for Health Statistics for the purpose of assessing the health and nutritional status of U.S. adults and children[14,15]. The survey samples are selected using a stratified multistage probability design with random sampling of the civilian non-institutionalized population, with over-sampling of certain subgroups. Our analyses were conducted with the approval of our Institutional Review Board.

Of the 21,004 individuals who participated in 1999-2002 surveys, we included 5,857 children and adolescents, ages 5 to 16 years. Adolescents' ages 17 years and older were not included in the study since dietary habits during this early post-high school period are often unstable due to significant lifestyle changes [16,17]. We excluded 539 subjects with missing or unreliable dietary data as well as 204 individuals with extreme energy intake (upper and lower 2% of the distribution). We further excluded subjects meeting any of the following criteria: missing CRP data (n = 590), pregnant or breastfeeding (n = 15), on dialysis or taking oral medications or insulin for diabetes (n = 11), missing data on covariates (n = 204), and those not able to be classified into one of primary racial groups (n = 107). Finally, to eliminate illness as a possible cause of elevated CRP, we excluded 77 subjects (mean CRP = 7.02 mg/L) whose white blood cell count was above the age- and sex-specific upper limit of normal as specified in the NHANES laboratory manuals[18]. The final study population included 4,110 children and adolescents.

### Assessment of food group intakes

From 1999 to 2001 dietary intake was assessed in NHANES with a single, multipass 24-hour recall, carried out using a computer-assisted dietary interview system that has been previously described[19]. A revised five step automated multiple-pass instrument was used starting in 2002[20]. Participants aged 12 years and older completed the interview independently. A proxy respondent responded for five-year olds while children ages 6 to11 years completed the interview together with a proxy respondent.

The dietary recalls were analyzed by the USDA using nutrient files that were appropriate to the time period of data collection[21,22]. Food Pyramid servings were estimated for each subject as defined by the USDA's Dietary Guidelines[23] (see additional file 1).

### Measurement of Highly Sensitive CRP (hs-CRP)

Blood samples were collected from all subjects, stored at -20 degrees centigrade and then shipped to the University of Washington Medical Center for analysis. The CRP concentration in serum was measured by a high-sensitivity assay, latex enhanced nephelometry, on a Dade Behring Nephelometer II Analyzer System. The analytical performance of this test has been validated[24] and shown to be comparable to that of ELISA[25].

### Potential confounders and modifiers of effect

We considered the following potential confounding variables in our analysis: exact age, race/ethnicity, height (in meters), height-for-age Z-Scores (HAZ), socioeconomic status (SES), body mass index (BMI) and BMI-Z Scores, waist circumference, sedentary behavior (hours of TV watching and video and computer time per day), energy intake (in kilo calories), percentage (%) of calories from carbohydrates, protein and fat and fiber intake per 1000 kilocalories of energy.

Racial/ethnic origin was classified into three categories: non-Hispanic whites, non-Hispanic blacks and Hispanics (i.e., Mexican-Americans). SES was estimated using combined data on income and food insecurity. Weight was measured on a Toledo Digital Scale and standing height (to the nearest 0.1 centimeter) using a fixed stadiometer. Waist circumference was measured with a steel tape (to the nearest 0.1 centimeter) at the high point of iliac crest at the end of normal expiration[26]. BMI was calculated as weight in kilograms divided by the square of height, in meters. CDC growth charts were used to calculate age and sex-specific BMI Z-scores and height-for-age Z-scores using the Lambda, Mu, Sigma (LMS) method[27].

### Statistical analysis

The goal of the analysis was to evaluate the association between serum CRP levels at 5 to16 years of age and dietary intake in the USDA food pyramid groups. Since NHANES provides only a single 24-hour dietary recall and single recalls may provide reliable estimates of population group means rather than individual means, we chose to estimate the adjusted food group means according to the child's CRP group assignment[28]. CRP was classified into one of three categories that have been previously shown to reflect low, average and high risk of future CVD[29]: <1.0 mg/L, 1.0-3.0 mg/L and >3.0 mg/L, respectively. Adjusted mean intakes of dairy, grains, fruit, vegetables and meat/other proteins were estimated in each of these categories using analysis of covariance modeling with Proc GLM.

All analyses were carried out using SAS, Version 9.1 (SAS Institute, Cary, NC). We used Proc Surveyreg to calculate adjusted mean differences in food group intake by CRP levels accounting for stratification and clustering within primary sampling units. Since we adjusted for the covariates representing the over-sampled subgroups, we did not adjust for sampling weights in our analysis; this adjustment is regarded as a good compromise between efficiency and bias[30]. The results of the Surveyreg Procedure were almost identical to the simpler GLM modeling, leading us to retain the GLM modeling in the results of this manuscript.

## Results

The characteristics of the study population in each of the three categories of CRP are summarized in Table 1. There were no meaningful differences in age or sedentary behavior across categories of CRP. The BMI (p < 0.0001), BMI Z-score (p < 0.0001) and waist circumference (p < 0.0001) increased with higher CRP levels. Children with low CRP levels were slightly shorter than those with higher levels. Non-Hispanic whites were most likely to be in the lowest CRP group while Hispanics were most likely to fall into the highest CRP category.

**Table 1:** Characteristics of children ages 5-16 years by C-reactive protein level in NHANES 1999-2002.

	**C-reactive protein levels (mg/L)**			
	**Low** **(<1.0)**	**Average** **(1.0-3.0)**	**High** **(>3.0)**	***P *value, two sided**
	**(n = 2939)**	**(n = 718)**	**(n = 453)**	
		
	**mean (SD)**			
C-reactive protein levels (mg/L)	0.29 (0.23)	1.8 (0.59)	8.6 (8.4)	-
				
Age (years)	11.4 (3.3)	11.6 (3.3)	11.6 (3.3)	*0.084*
				
Height (inches)	58.8 (7.6)	59.4 (7.3)	59.5 (7.2)	*0.022*
				
Height-for-age Z-Score	0.12 (1.1)	0.21 (1.1)	0.27 (1.0)	*0.002*
				
BMI (Kg/m^2^)	19.6 (4.1)	23.8 (5.9)	25.2 (7.7)	*<0.0001*
				
BMI Z-score	0.29 (1.0)	1.2 (1.0)	1.3 (1.2)	*<0.0001*
				
Waist circumference (cms)	68.8 (12.1)	79.4 (16.5)	83.3 (20.4)	*<0.0001*
				
Sedentary behavior (hrs/day)*	2.9 (1.5)	2.9 (1.6)	3.0 (1.5)	*0.728*
				
Food groups (servings/day)				
Dairy	2.0 (1.6)	1.8 (1.4)	1.9 (1.6)	*0.012*
Grains	7.1 (3.8)	6.8 (3.6)	6.3 (3.5)	*<0.0001*
Fruit	1.5 (2.0)	1.3 (1.6)	1.4 (1.9)	*0.384*
Vegetables	2.6 (2.4)	2.4 (2.1)	2.2 (2.2)	*0.002*
Meat/Other Proteins	4.0 (3.2)	4.0 (3.2)	3.9 (3.1)	*0.380*
				
Energy intake (kcal)	2103.5 (14.7)	2011.9 (29.8)	1930.4 (37.5)	*<0.0001*
				
Energy adjusted macronutrients				
% calories from fat	32.8 (7.5)	32.7 (8.2)	32.2 (8.4)	*0.213*
% calories from carbohydrates	54.7 (9.3)	54.6 (10.0)	54.8 (11.0)	*0.917*
% calories from protein	13.7 (3.9)	13.8 (4.1)	14.1 (4.4)	*0.064*
fiber intake/1000 kcal of energy	6.4 (2.9)	6.3 (2.8)	6.2 (3.1)	*0.163*
				
	No. (row percent)			
Gender				
Girls	1435 (69.9)	373 (18.2)	245 (11.9)	*0.057*
Boys	1504 (73.1)	345 (16.8)	208 (10.1)	
				
Race/Ethnicity				*<0.0001*
Non-Hispanic Whites	885 (78.7)	144 (12.8)	96 (8.5)	
Non-Hispanic Blacks	922 (72.0)	227 (17.7)	131 (10.2)	
Hispanic	1132 (66.4)	347 (20.4)	226 (13.3)	
				
Socioeconomic Status				*0.006*
Low	997 (69.1)	259 (17.9)	188 (13.0)	
High	1942 (72.8)	459 (17.2)	265 (9.9)	

Abbreviations: BMI, body mass index; NHANES, *National Health and Nutrition Examination Survey; *SD, standard deviation; kcal, kilocalories of energy

* The number of usual hours spent watching television, playing video games or using a computer

CRP levels were inversely associated with energy intake (p < 0.0001); those in highest CRP group reported consuming the fewest calories. To address the concern of biased reporting of energy intake, we also examined the association between energy intake and BMI. We found that those in the highest tertile of BMI consumed fewer calories (2014.2 kcal) than those in middle (2079.3 kcal) and lower (2112.0 kcal) tertiles (data not shown).

The adjusted mean food group intakes in the three categories of CRP are shown in Table 2. After adjusting for age, gender, race/ethnicity, height, SES, and sedentary behavior, children in the lowest CRP category consumed more grains (p < 0.001), vegetables (p = 0.0002) and dairy (p = 0.047). We also explored controlling for the height-for-age Z-scores and found nearly identical results, leading us to retain the original height variable in the models (see additional file 2).

**Table 2:** Adjusted mean food intakes according to C-reactive protein level in children ages 5-16 years

C-reactive protein levels (mg/L)*	***n***	**Dairy**	**Grains**	**Fruit**	**Vegetables**	**Meat/Other Proteins**
		
		**mean (SE)**				
	Baseline analysis^†^					
Low	2939	1.98 (0.03)	7.09 (0.07)	1.46 (0.04)	2.59 (0.04)	4.05 (0.06)
Average	718	1.81 (0.06)	6.81 (0.14)	1.31 (0.07)	2.41 (0.08)	3.98 (0.12)
High	453	1.90 (0.07)	6.35 (0.17)	1.41 (0.09)	2.19 (0.11)	3.88 (0.15)
*P for trend*		*0.047*	*<.0001*	*0.244*	*0.0002*	*0.249*
						
	Body composition					
	*Adding BMI Z-Score to the baseline model*					
Low	2939	1.96 (0.03)	7.04 (0.07)	1.44 (0.04)	2.58 (0.04)	4.06 (0.06)
Average	718	1.84 (0.06)	6.92 (0.14)	1.34 (0.07)	2.43 (0.09)	3.96 (0.12)
High	453	1.93 (0.07)	6.48 (0.18)	1.46 (0.09)	2.21 (0.11)	3.86 (0.15)
*P for trend*		*0.304*	*0.006*	*0.751*	*0.001*	*0.199*
	*Adding Waist Circumference to the baseline model*^‡^					
Low	2922	1.95 (0.03)	7.02 (0.07)	1.44 (0.04)	2.59 (0.04)	4.05 (0.06)
Average	713	1.87 (0.06)	6.96 (0.14)	1.35 (0.08)	2.41 (0.09)	3.97 (0.12)
High	450	1.97 (0.07)	6.59 (0.18)	1.49 (0.10)	2.18 (0.11)	3.88 (0.15)
*P for trend*		*0.770*	*0.057*	*0.925*	*0.001*	*0.277*
						
	Other dietary factors					
	*Adding other dietary food groups to the baseline model*					
Low	2939	1.96 (0.03)	7.06 (0.07)	1.45 (0.04)	2.58 (0.04)	4.02 (0.06)
Average	718	1.83 (0.05)	6.90 (0.13)	1.32 (0.07)	2.43 (0.08)	4.01 (0.11)
High	453	1.95 (0.07)	6.40 (0.17)	1.45 (0.09)	2.22 (0.11)	4.00 (0.14)
*P for trend*		*0.309*	*0.001*	*0.521*	*0.001*	*0.839*
	*Adding percent calories from fat to baseline model*					
Low	2939	1.98 (0.03)	7.09 (0.07)	1.46 (0.04)	2.59 (0.04)	4.04 (0.05)
Average	718	1.81 (0.06)	6.81 (0.14)	1.31 (0.07)	2.41 (0.08)	3.98 (0.11)
High	453	1.91 (0.07)	6.34 (0.17)	1.39 (0.09)	2.21 (0.11)	3.93 (0.14)
*P for trend*		*0.059*	*<.0001*	*0.152*	*0.0004*	*0.394*
	*Adding percent of calories from carbohydrates to baseline model*					
Low	2939	1.98 (0.03)	7.09 (0.07)	1.46 (0.03)	2.59 (0.04)	4.05 (0.05)
Average	718	1.81 (0.06)	6.81 (0.14)	1.31 (0.07)	2.41 (0.08)	3.98 (0.10)
High	453	1.90 (0.07)	6.35 (0.17)	1.41 (0.09)	2.19 (0.11)	3.90 (0.13)
*P for trend*		*0.049*	*<.0001*	*0.212*	*0.0002*	*0.233*
	*Adding percent of calories from proteins to baseline model*					
Low	2939	1.98 (0.03)	7.08 (0.07)	1.46 (0.04)	2.59 (0.04)	4.07 (0.05)
Average	718	1.81 (0.05)	6.81 (0.14)	1.31 (0.07)	2.41 (0.08)	3.98 (0.09)
High	453	1.88 (0.07)	6.39 (0.17)	1.42 (0.09)	2.19 (0.11)	3.75 (0.12)
*P for trend*		*0.022*	*<.0001*	*0.287*	*0.0002*	*0.011*
	*Adding Fiber intake/1000 kilocalories of energy to baseline model*					
Low	2939	1.98 (0.03)	7.09 (0.07)	1.45 (0.03)	2.57 (0.04)	4.07 (0.06)
Average	718	1.81 (0.06)	6.82 (0.14)	1.32 (0.07)	2.44 (0.08)	3.96 (0.11)
High	453	1.89 (0.07)	6.36 (0.17)	1.45 (0.09)	2.25 (0.10)	3.84 (0.14)
*P for trend*		*0.031*	*<.0001*	*0.520*	*0.002*	*0.127*

Abbreviations: SE, standard error;

*CRP levels: Low = <1.0 mg/L, Average = 1.0-3.0 mg/L, High = >3.0 mg/L

^†^Adjusted for age, gender, race/ethnicity, height, socioeconomic status, and sedentary behavior. ^‡^25 subjects were missing information on waist circumference

Effect modification was assessed using a product term in the multivariable models. The only statistically significant interactions were found between age and CRP levels (for grain intake) (p = 0.03) and for race/ethnicity and CRP levels for meat intake (p = 0.02). In that model, there was an inverse association between CRP and meat intake among Hispanics (p = 0.021) but no association was observed among Non-Hispanic whites (p = 0.617) and Non-Hispanic blacks (p = 0.792) (see additional file 3).

### Exploring role of body composition and other dietary factors

The weak but beneficial association between CRP level and dairy consumption in the baseline analysis was attenuated by controlling for either BMI Z-score (p for linear trend changed from p = 0.047 to p = 0.304) or waist circumference (changed from p = 0.047 to p = 0.770). There was only a small amount of attenuation of the associations of grains and vegetables with CRP level. In the final analysis in this table, all other food groups were added to the baseline multivariable models. In this analysis, only an independent association remained for grains and vegetables with CRP. In contrast, the inclusion of energy-adjusted macronutrients and fiber to the models did not fully attenuate the observed associations between any of the food groups and CRP in these children.

The associations of food subgroups and CRP levels are shown in Table 3. A number of the food subgroups here are consumed infrequently by these children and adolescents (e.g., yogurt, whole grains, dark green/leafy vegetables, deep yellow/orange vegetables), giving us little statistical power to examine their relationship with CRP. The milk subgroup was inversely associated with CRP (p = 0.040) while cheese consumption was not. Refined grain intake appeared to be beneficially associated with CRP (p < 0.0001). Other food subgroups found to be beneficially related to CRP level included citrus, melons, and berries (p = 0.053), tomatoes (p = 0.001), total non-starchy vegetables (p = 0.004), and total starchy vegetables (p = 0.012). Neither total meat nor any of the meat/other protein subgroups were associated with CRP levels.

**Table 3:** Adjusted mean food subgroups intakes according to C-reactive protein level in children ages 5-16 years

	**C-reactive protein levels (mg/L)**			
	**Low** **(<1.0)**	**Average** **(1.0-3.0)**	**High** **(>3.0)**	***P for trend***
	**(n = 2939)**	**(n = 718)**	**(n = 453)**	
	
	mean (SE)*			
Dairy (total)	1.98 (0.03)	1.81 (0.06)	1.90 (0.07)	*0.047*
Milk	1.36 (0.02)	1.26 (0.05)	1.26 (0.06)	*0.040*
Cheese	0.59 (0.01)	0.52 (0.03)	0.62 (0.04)	*0.838*
Yogurt	0.02 (0.002)	0.02 (0.005)	0.01 (0.01)	*0.406*
				
Grains (total)	7.09 (0.07)	6.81 (0.14)	6.35 (0.17)	*<.0001*
Refined grains	6.36 (0.06)	6.09 (0.13)	5.70 (0.16)	*<.0001*
Whole Grains	0.73 (0.02)	0.72 (0.05)	0.65 (0.06)	*0.258*
				
Fruit (total)	1.46 (0.04)	1.31 (0.07)	1.41 (0.09)	*0.244*
Citrus, melon, berries	0.70 (0.03)	0.63 (0.05)	0.58 (0.07)	*0.053*
Other fruit	0.76 (0.02)	0.67 (0.05)	0.83 (0.06)	*0.707*
				
Vegetables (total)	2.59 (0.04)	2.41 (0.08)	2.19 (0.11)	*0.0002*
Total Non-Starchy (Green, Yellow, Tomatoes and Others)	1.19 (0.02)	1.07 (0.05)	1.03 (0.06)	*0.004*
Dark green	0.07 (0.01)	0.07 (0.01)	0.06 (0.02)	*0.461*
Deep yellow/orange	0.09 (0.01)	0.08 (0.01)	0.06 (0.02)	*0.104*
Tomatoes	0.50 (0.01)	0.41 (0.03)	0.42 (0.03)	*0.001*
Other vegetables	0.53 (0.02)	0.52 (0.03)	0.49 (0.04)	*0.295*
Total Starchy (Potatoes, legumes & Others)	1.40 (0.03)	1.34 (0.07)	1.16 (0.08)	*0.012*
Legumes	0.20 (0.01)	0.18 (0.02)	0.15 (0.03)	*0.090*
Potatoes	1.07 (0.03)	1.05 (0.06)	0.91 (0.08)	*0.079*
Other Starchy Vegetables	0.12 (0.01)	0.11 (0.02)	0.10 (0.02)	*0.212*
				
Meat/Other Proteins	4.05 (0.06)	3.98 (0.12)	3.88 (0.15)	*0.249*
Red meat	1.52 (0.04)	1.50 (0.08)	1.55 (0.10)	*0.952*
White meat (poultry, fish, other seafood)	1.34 (0.04)	1.32 (0.08)	1.20 (0.10)	*0.258*
Organ meats	0.01 (0.004)	0.005 (0.01)	0.01 (0.01)	*0.308*
Processed meats	0.74 (0.02)	0.70 (0.05)	0.73 (0.06)	*0.626*
Other protein sources (Eggs, Nuts, Seeds and Soy)	0.43 (0.02)	0.46 (0.03)	0.39 (0.04)	*0.610*

Abbreviations: SE, standard error;

*Adjusted for age, gender, race/ethnicity, height, socioeconomic status, and sedentary behavior.

## Discussion

This study found that children and adolescents with higher CRP levels had significantly lower intakes of grains and vegetables. There was also a weak beneficial association between dairy intake and CRP level, particularly for milk consumption. The low intakes of a number of food subgroups made it difficult to assess the associations of these dietary factors with CRP. Certain subgroups such as tomatoes and citrus, melon and berries were found to be beneficially associated with CRP. Both starchy and non-starchy vegetables had similar associations with CRP.

In general, the addition of body composition variables to the multivariable models attenuated the associations between food consumption pattern and CRP levels. Only the intakes of grains and vegetables were independent of BMI and waist circumference. The mechanism by which different foods may affect the sub-clinical inflammatory state is not clear although these results do suggest that body composition plays a role.

Obesity is a potent risk factor for the development of atherosclerosis as well as non-insulin dependent diabetes mellitus and these effects may be due in part to the sub-clinical inflammatory response associated with excess adipose tissue[31]. It has been reported that adipose tissue secrete cytokines such as IL-6 that in turn regulate CRP production[32]. Both obesity and inflammation are related in a circular fashion. Excessive weight gain stimulates an inflammatory response while inflammation itself leads to greater weight gain[33]. Recent published studies in children indicate that excess adiposity is associated with significantly higher levels of CRP[34,35] and several other studies suggest that high CRP levels may lead to early atherosclerotic changes in children[36].

The association between fruit and vegetable consumption and CRP in this study is consistent with some earlier studies of adults. Higher intakes of citrus, melon, berries or tomatoes may be linked with healthier lifestyles in general and with beneficial effects on body fat acquisition. Fruits and vegetables are low-energy dense foods (fewer calories per gram of food) with high contents of water and fiber. The larger volume of food consumed combined with the low energy density may benefit satiety[37], energy balance and weight status[38]. While controlling for body composition in our analyses attenuated the effects of fruit intake, the association between vegetable consumption and CRP was unchanged, suggesting that the effects of vegetables in this study was not entirely explained by differences in body composition.

Some food groups such as vegetables and fruits are particularly rich in antioxidant compounds including vitamin C[39], beta carotene[40], dietary flavonoids[41], and lycopene[42]. These anti-oxidants may lower the concentration of CRP, thereby reducing sub-clinical inflammation. In one study, the total antioxidant capacity of the diet was shown to be independently associated with lower CRP levels, leading the authors to suggest that this mechanism might be part of the explanation for the beneficial effects of certain dietary factors such as fruits, whole cereals and red wine on the risk of CVD [43]. In addition, low levels of antioxidant vitamin intake have also been associated with obesity in children[44].

Unlike vegetables, the association between CRP levels and dairy consumption in this study was attenuated by the inclusion of either BMI or waist circumference in the multivariable models. Thus, it is possible that the association between dairy and CRP is explained by previously shown beneficial effects of dairy intake on body composition[45]. Dairy intake may also be linked with greater satiety or a healthier diet in general. In a recent study in mice, a diet rich in dairy led to decreases in the expression of several inflammatory cytokine genes in adipose tissue[46] which was in turn followed by decreases in the levels of plasma IL-6 and its associated inflammatory marker CRP.

The inverse association between CRP and grain intake was not explained by differences in body composition. Neither body composition nor the intake of macronutrients or other foods explained the relation between CRP and refined grains. The CRP-grain association in this study is consistent with findings among adults in NHANES-III[47] but not consistent with effects found in other studies[48]. In one recent study of adults on a hypo-caloric diet, the intake of both whole and refined grains was associated with moderate weight loss but only whole grain consumption led to reductions in CRP levels[49].

There are several limitations of this study, the most important of which is its cross-sectional design. Certainly, it is very unlikely that the reported dietary intake in this study is biased by knowledge of CRP level. However, it is possible that the reported dietary intakes are biased by the level of body fat which is closely linked with CRP. This bias is more likely present among adolescents than among younger children.

A second important limitation of this study is the availability of only a single 24 hour dietary recall, thereby providing a less precise measure of intake. The use of a single recall does not introduce bias but will reduce the power to detect true effects that may exist, especially in population subgroups (e.g., stratifying by race/ethnicity) and for infrequently-eaten foods. To minimize error associated with using a single 24-hour recall to classify children according to dietary intake, we classified the children according to CRP level and examined mean dietary intakes in these groups[28].

We were unable to exclude certain chronic diseases or prevalent acute infections in the children. However, we did exclude those subjects whose white blood cell count was above the upper limit of the reference range from the NHANES laboratory procedural manual. Another limitation of this analysis is that we were unable to control for physical activity since these data were not collected for children at ages 5-11 years of age. While the association between physical activity and CRP levels during childhood is uncertain[50], we attempted to address this potential confounding issue by including a measure of sedentary behavior in the multivariable models.

There are certain strengths of this study that we would like to mention. NHANES is a comprehensive national survey with a large sample size (and over-sampling of minorities), making the results more generalizable to US population. To our knowledge this is the first study to examine the effects of USDA food group intakes on CRP levels in children, while examining the role of number of potential modifiers of this relationship.

## Conclusion

In conclusion, in this cross-sectional study, higher levels of CRP were associated with lower intakes of vegetables and grains and, to a lesser degree, lower intakes of dairy. Only the associations seen with grains and vegetable intakes were independent of body composition. Higher intakes of certain food subgroups, such as milk, citrus, melons and berries, and tomatoes were also associated with lower levels of CRP. These dietary relationships with CRP should be further examined in future prospective studies.

## Abbreviations

**CRP**: C-reactive protein; **NHANES**: National Health and Nutrition Examination Surveys; **BMI**: Body mass index; **IL-6**: Interleukin-6; **hs-CRP**: highly sensitive CRP assays; **CVD**: cardiovascular diseases; **LD**L: low-density lipoprotein; **USDA**: United States Department of Agriculture; **SES**: socioeconomic status; **ELISA**: Enzyme-linked immunosorbent assay; **CDC**: Centers for Disease Control and Prevention; **LMS**: lambda, Mu, Sigma; **TAC**: Total antioxidant capacity; **p**: *p for trend*

## Competing interests

Each of the authors has read and approved the final version of the manuscript and takes responsibility for its content. None of the authors has any conflict of interest that would potentially influence this work

## Authors' contributions

MMQ helped with conception and design of the analysis, carried out data analyses, participated in the interpretation of the results and wrote the manuscript.

MRS was the study's nutritionist, participating in the conception of the study, preparation of data sets for analysis and aiding in the editing of the manuscript.

LLM supervised and participated in all aspects of the study design, analysis, interpretation, and preparation of the manuscript.

## Supplementary Material

Additional file 1 **Description of food pyramid groups in NHANES 1999-2002**. The table outlines food pyramid groups according to USDA's Dietary Guidelines. Click here for file

Additional file 2 **Adjusted mean food intakes according to C-reactive protein level in children ages 5-16 years: Exploring Height-for-age Z-scores**. The data provided present results from an analysis where we replaced height (inches) with Height-for-age Z-scores in our baseline model. Click here for file

Additional file 3 **Adjusted mean food intakes according to C-reactive protein level in children ages 5-16 years: Exploring effect modification by age, gender and race/ethnicity.** The file contains results for the effect modification analysis Click here for file
