# Supplementary material for: A cross-sectional study of food group intake and C-reactive protein among children
Source: Nutr Metab (Lond). 2009 Oct 12;6:40. doi: 10.1186/1743-7075-6-40 (PMC2770558; doi:10.1186/1743-7075-6-40)
Supplement: Additional file 1 — Description of food pyramid groups in NHANES 1999-2002. The table outlines food pyramid groups according to USDA's Dietary Guidelines. [file 1743-7075-6-40-S1.DOC]

| **Description of food pyramid groups in NHANES 1999-2002.** | | | | |
| --- | --- | --- | --- | --- |
| Dairy Group | Grains Group | Meat and Other Proteins Group | Fruit Group | Vegetable Group |
| The dairy group includes milk, cheese and yogurt.  One serving of dairy is defined as a cup of milk or yogurt, 1.5-2.0 ounces of dry cheese, and 1 ounce of low-fat cheese. For other cheeses (e.g., cottage cheese, cream cheese), one serving is the amount that provides the same amount of calcium as a cup of milk. | Grains include foods made from wheat, rice, barley, rye and oats such as bread, crackers, dry and cooked cereals. Grains are subdivided as whole or refined grain products.  One serving equals a slice of bread; one-half cup of cooked cereal, rice, or pasta; or about 1 cup of dry cereal. | The *Dietary Guidelines* “meat” group includes both meats and other non-dairy proteins such as red meat, white meat (poultry, fish, and other seafood), organ meats, processed meats, eggs, nuts, seeds, and soy products.  In general, a serving is a “one-ounce meat equivalent” that corresponds with the amount of protein in one ounce of lean meat (e.g., one egg, two tablespoons of peanut butter, 1/3 cup of nuts, ¼ cup of seeds, or ½ cup of tofu). | Total fruit intake (whole or as juice) is divided into two subgroups: a) citrus, melon, and berries and b) other fruits. The citrus, melon and berries group includes various citrus fruits, berries, melons, cantaloupe, and other similar fruits. “Other fruits” included fruits such as apples, bananas, pears or grapes.  One fruit serving comprises such items as whole fruit (an apple or banana), a grapefruit half, melon wedges, ¾ cup of fruit juice, ½ cup berries, ½ cup chopped, cooked, or canned fruit, and ¼ cup of dried fruit. | Vegetables are further classified as follows: a) dark-green vegetables such as lettuce, spinach, grape leaves, b) deep-yellow/orange vegetables such as carrots and pumpkins, c) tomatoes, d) other vegetables as cabbage, onions and peppers, e) potatoes, f) legumes and g) other starchy vegetables such as peas and corn.  One vegetable serving includes one cup of raw leafy vegetables, ½ cup of other vegetables (raw or cooked), or ¾ cup of vegetable juice. |
