# Supplementary material for: A cross-sectional study of food group intake and C-reactive protein among children
Source: Nutr Metab (Lond). 2009 Oct 12;6:40. doi: 10.1186/1743-7075-6-40 (PMC2770558; doi:10.1186/1743-7075-6-40)
Supplement: Additional file 2 — Adjusted mean food intakes according to C-reactive protein level in children ages 5-16 years: Exploring Height-for-age Z-scores. The data provided present results from an analysis where we replaced height (inches) with Height-for-age Z-scores in our baseline model. [file 1743-7075-6-40-S2.DOC]

| **Adjusted mean food intakes according to C-reactive protein level in children ages 5-16 years: Exploring Height-for-age Z-scores** | | | | | | |
| --- | --- | --- | --- | --- | --- | --- |
| C-reactive protein levels  (mg/L) | *n* | Dairy | Grains | Fruit | Vegetables | Meat/Other Proteins |
|  |  | mean (SE)**^*^** | | | | |
| Low (<1.0) | 2939 | 1.98 (0.03) | 7.09 (0.07) | 1.46 (0.04) | 2.59 (0.04) | 4.05 (0.06) |
| Average (1.0-3.0) | 718 | 1.81 (0.06) | 6.81 (0.14) | 1.30 (0.07) | 2.41 (0.08) | 3.98 (0.12) |
| High (>3.0) | 453 | 1.89 (0.07) | 6.35 (0.17) | 1.40 (0.09) | 2.19 (0.11) | 3.87 (0.15) |
| *p for trend* |  | *0.040* | *<0.0001* | *0.189* | *0.0002* | *0.230* |

^*^Adjusted for age, gender, race/ethnicity, height-for-age Z-scores, socio-economic status, and sedentary behavior
