# Supplementary material for: A cross-sectional study of food group intake and C-reactive protein among children
Source: Nutr Metab (Lond). 2009 Oct 12;6:40. doi: 10.1186/1743-7075-6-40 (PMC2770558; doi:10.1186/1743-7075-6-40)
Supplement: Additional file 3 — Adjusted mean food intakes according to C-reactive protein level in children ages 5-16 years: Exploring effect modification by age, gender and race/ethnicity. The file contains results for the effect modification analysis [file 1743-7075-6-40-S3.DOC]

| **Adjusted mean food intakes according to C-reactive protein level in children ages 5-16 years: Exploring effect modification by age, gender and race/ethnicity** | | | | | | |
| --- | --- | --- | --- | --- | --- | --- |
| C-reactive protein levels (mg/L)^*^ | *n* | Dairy | Grains | Fruit | Vegetables | Meat/Other  Proteins |
|  |  | mean (SE) | | | | |
|  | Baseline Analysis^†^ | | | | | |
| Low | 2939 | 1.98 (0.03) | 7.09 (0.07) | 1.46 (0.04) | 2.59 (0.04) | 4.05 (0.06) |
| Average | 718 | 1.81 (0.06) | 6.81 (0.14) | 1.31 (0.07) | 2.41 (0.08) | 3.98 (0.12) |
| High | 453 | 1.90 (0.07) | 6.35 (0.17) | 1.41 (0.09) | 2.19 (0.11) | 3.88 (0.15) |
| *p for trend* |  | *0.047* | *<.0001* | *0.244* | *0.0002* | *0.249* |
|  |  |  |  |  | | |
|  | Stratified by Age^†^ | | | | | |
|  | *Children, Ages 5-11* | | | | | |
| Low | 1294 | 2.08 (0.04) | 6.83 (0.09) | 1.62 (0.05) | 2.34 (0.06) | 3.71 (0.08) |
| Average | 306 | 1.93 (0.08) | 6.72 (0.19) | 1.40 (0.11) | 2.23 (0.11) | 3.64 (0.16) |
| High | 199 | 1.88 (0.10) | 6.05 (0.23) | 1.51 (0.14) | 1.92 (0.14) | 3.48 (0.19) |
| *p for trend* |  | *0.028* | *0.005* | *0.168* | *0.007* | *0.265* |
|  | *Adolescents, Ages 12-16* | | | | | |
| Low | 1645 | 1.90 (0.04) | 7.31 (0.10) | 1.34 (0.05) | 2.79 (0.06) | 4.33 (0.08) |
| Average | 412 | 1.72 (0.08) | 6.85 (0.20) | 1.22 (0.09) | 2.54 (0.12) | 4.21 (0.16) |
| High | 254 | 1.90 (0.10) | 6.56 (0.25) | 1.31 (0.12) | 2.39 (0.16) | 4.16 (0.21) |
| *p for trend* |  | *0.395* | *0.001* | *0.539* | *0.006* | *0.378* |
|  |  |  |  |  | | |
|  | Stratified by Gender^‡^ | | | | | |
|  | *Girls* | | | | | |
| Low | 1435 | 1.72 (0.03) | 6.36 (0.08) | 1.45 (0.05) | 2.44 (0.05) | 3.57 (0.07) |
| Average | 373 | 1.58 (0.07) | 6.03 (0.17) | 1.26 (0.10) | 2.20 (0.11) | 3.41 (0.14) |
| High | 245 | 1.74 (0.08) | 5.60 (0.21) | 1.59 (0.13) | 2.07 (0.13) | 3.42 (0.18) |
| *p for trend* |  | *0.642* | *0.0003* | *0.873* | *0.002* | *0.278* |

|  | *Boys* | | | | | |
| --- | --- | --- | --- | --- | --- | --- |
| Low | 1504 | 2.24 (0.04) | 7.82 (0.11) | 1.46 (0.05) | 2.74 (0.06) | 4.53 (0.09) |
| Average | 345 | 2.03 (0.09) | 7.57 (0.22) | 1.37 (0.10) | 2.61 (0.13) | 4.53 (0.18) |
| High | 208 | 2.05 (0.11) | 7.14 (0.28) | 1.21 (0.13) | 2.32 (0.17) | 4.35 (0.23) |
| *P for trend* |  | *0.029* | *0.022* | *0.075* | *0.022* | *0.538* |
|  |  |  |  |  | | |
|  | Stratified by Race/Ethnicity^§^ | | | | | |
|  | *Non-Hispanic Whites* | | | | | |
| Low | 885 | 2.37 (0.06) | 7.31 (0.12) | 1.41 (0.07) | 2.42 (0.08) | 3.58 (0.10) |
| Average | 144 | 1.99 (0.15) | 6.73 (0.31) | 1.28 (0.17) | 2.20 (0.19) | 3.96 (0.26) |
| High | 96 | 2.29 (0.18) | 6.57 (0.38) | 1.07 (0.21) | 2.04 (0.23) | 3.53 (0.32) |
| *P for trend* |  | *0.161* | *0.019* | *0.104* | *0.075* | *0.617* |
|  | *Non-Hispanic Blacks* | | | | | |
| Low | 922 | 1.55 (0.04) | 6.93 (0.12) | 1.34 (0.06) | 2.51 (0.07) | 4.49 (0.10) |
| Average | 227 | 1.45 (0.08) | 6.52 (0.24) | 1.35 (0.12) | 2.39 (0.15) | 4.40 (0.21) |
| High | 131 | 1.56 (0.10) | 6.60 (0.32) | 1.53 (0.16) | 2.27 (0.19) | 4.63 (0.28) |
| *P for trend* |  | *0.641* | *0.149* | *0.324* | *0.200* | *0.792* |
|  | *Hispanics* | | | | | |
| Low | 1132 | 2.03 (0.04) | 7.05 (0.11) | 1.58 (0.06) | 2.76 (0.07) | 4.05 (0.09) |
| Average | 347 | 1.91 (0.08) | 7.02 (0.20) | 1.33 (0.10) | 2.55 (0.12) | 3.75 (0.16) |
| High | 226 | 1.91 (0.10) | 6.11 (0.24) | 1.52 (0.13) | 2.27 (0.15) | 3.61 (0.20) |
| *P for trend* |  | *0.147* | *0.002* | *0.258* | *0.002* | *0.021* |
|  |  |  |  |  |  |  |

Abbreviations: SE, standard error

^*^CRP levels: Low= <1.0 mg/L, Average= 1.0-3.0 mg/L, High= >3.0 mg/L

^†^Adjusted for age, gender, race/ethnicity, height, socio-economic status, and sedentary behavior. ^‡^Dropping gender from the model. ^§^Dropping race/ethnicity from the model.
